# Improving Structure Delineation for Radiation Therapy Planning Using Dual-Energy CT

**DOI:** 10.3389/fonc.2020.01694

**Published:** 2020-08-28

**Authors:** George Noid, Justin Zhu, An Tai, Nilesh Mistry, Diane Schott, Douglas Prah, Eric Paulson, Christopher Schultz, X. Allen Li

**Affiliations:** ^1^Department of Radiation Oncology, Medical College of Wisconsin, Milwaukee, WI, United States; ^2^Siemens Medical Solutions USA, Inc., Malvern, PA, United States

**Keywords:** dual-energy CT, radiation oncology, radiation therapy, image contrast, metal artifacts, motion artifacts, photon starvation, mono-energetic image

## Abstract

**Purpose:**

We present the advantages of using dual-energy CT (DECT) for radiation therapy (RT) planning based on our clinical experience.

**Methods:**

DECT data acquired for 20 representative patients of different tumor sites and/or clinical situations with dual-source simultaneous scanning (Drive, Siemens) and single-source sequential scanning (Definition, Siemens) using 80 and 140-kVp X-ray beams were analyzed. The data were used to derive iodine maps, fat maps, and mono-energetic images (MEIs) from 40 to 190 keV to exploit the energy dependence of X-ray attenuation. The advantages of using these DECT-derived images for RT planning were investigated.

**Results:**

When comparing 40 keV MEIs to conventional 120-kVp CT, soft tissue contrast between the duodenum and pancreatic head was enhanced by a factor of 2.8. For a cholangiocarcinoma patient, contrast between tumor and surrounding tissue was increased by 96 HU and contrast-to-noise ratio was increased by up to 60% for 40 keV MEIs compared to conventional CT. Simultaneous dual-source DECT also preserved spatial resolution in comparison to sequential DECT as evidenced by the identification of vasculature in a pancreas patient. Volume of artifacts for five patients with titanium implants was reduced by over 95% for 190 keV MEIs compared to 120-kVp CT images. A 367-cm^3^ region of photon starvation was identified by low CT numbers in the soft tissue of a mantle patient in a conventional CT scan but was eliminated in a 190 keV MEI. Fat maps enhanced image contrast as demonstrated by a meningioma patient.

**Conclusion:**

The use of DECT for RT simulation offers clinically meaningful advantages through improved simulation workflow and enhanced structure delineation for RT planning.

## Introduction

Conventional poly-energetic CT is the most widely used imaging modality for radiation therapy (RT) planning. CT data are used for defining target volumes and distinguishing them from surrounding organs at risk (OAR). The study sets are subsequently utilized for calculating treatment plans, from which dose volume histogram (DVH) statistics are obtained to quantify the plan’s effectiveness relative to the prescription, with the goal of adequately covering the targets while maximally sparing OARs. Hence, RT planning depends on the quality of the planning images, which may lack in image contrast and functional information due to the limitations of poly-energetic CT (1).

A typical solution to the problem of poor image contrast is to register the CT with other imaging modalities (MRI and PET) to offer enhanced image contrast and information about functional or metabolic activity (2). However, such registration inherently introduces registration uncertainty, which may be substantial in some cases due to differences in patient setup (3). Furthermore, information from other imaging modalities may not be available for some patients.

In addition, metal implants cause artifacts (4) and/or photon starvation (5) due to high attenuation in conventional CT images. Metal artifacts and uncorrected photon starvation in the clinic produces aberrant CT numbers (CTNs) that can deteriorate the patient’s whole body contour (4), necessitating the use of manual rather than auto segmentation (6). In addition to increasing the workload, a manually generated patient contour is inherently subjective, thus introducing uncertainty into the treatment plan.

Dual-energy CT (DECT) is an emerging technology in radiation oncology that can help address these problems (7). It involves the acquisition of two poly-energetic CT study sets with different energy spectra either simultaneously (e.g., dual-source, fast switching, partial spectral filtering) (7) or sequentially (e.g., dual spiral) (8). These two acquisitions can be used to derive a variety of images that can address the deficiencies in conventional CT. These images include virtual mono-energetic images (MEIs) (9) and material decompositions, e.g., iodine maps (I maps) (10), fat maps (11), effective Z maps (12), maps of lung perfusion blood volume (13), and relative electron density images (14). Lung perfusion blood volume maps are a subset of iodine maps that use color enhancement to highlight iodinated areas of the lungs, thus allowing for visualization of perfused blood volume. These derived images can enhance soft tissue contrast, restore voxels that are lost due to image artifacts or photon starvation, assist in the delineation of vasculature, and aid in visualizing lung function (9, 15). The derived images provide additional information without introducing registration uncertainty when they are calculated from simultaneous DECT acquisitions.

In poly-energetic CT, the CTNs are averaged over the entire X-ray energy spectrum, which is typically characterized by the maximum tube potential (e.g., 120-kVp), whereas the mean energy is closer to 70 keV (7). By averaging over the entire energy spectrum, useful information encoded in the lower energy end [i.e., Rayleigh Scattering and the photo-electric effect (16)] is obscured. MEIs derived from DECT can uncover this information and enhance soft tissue contrast (17). A recent study found that lower-energy MEIs provided better signal-to-noise ratios compared to a simulated linearly blended 120-kVp set for pancreatic cancer cases (18). Another study corroborated this observation by comparing sequential DECT (SE-DECT) MEIs to conventional 120-kVp CT (19).

In addition, a recent study by Zhou et al. (9) found that higher-energy MEIs can significantly reduce metal artifacts. A similar study by Yue et al. (20) corroborated this, noting that higher-energy MEIs combined with metal artifact-reducing algorithms can further reduce metal artifacts. Furthermore, it is possible to perform multi-material decompositions based on the CTNs from the two acquisitions of a DECT, enabling the quantification of the presence of iodine, fat, or other constituents of a given voxel (21).

Several solutions exist for implementing DECT in the clinic. Sequential protocols offer the lowest technical barriers and are comparatively cost-effective (22). The primary limitation of a SE-DECT protocol is that the image quality of the derived images is affected by patient motion between the two scans (23). The two acquisitions are separated in time by 5–10 s (depending on the scan length, rotation time, and pitch), which can cause a registration mismatch of the two scans that greatly degrades image quality, increases noise, and decreases spatial resolution (23).

Simultaneous DECT protocols vastly reduce the impact of patient-related motion and motion-related noise, thus enhancing spatial resolution (24). Therefore, simultaneous DECT can be applied to most patient cases, unlocking its potential across the practice of radiation oncology. Several solutions exist for performing simultaneous DECT, including dual-source DECT (DS-DECT) (4), fast-switching X-ray tubes (25), multilayer detectors (26), and photon counting detectors (23). DS-DECT features two tubes and detector systems in the same gantry (4). A tin filter can be used on the higher energy tube to increase the spectral separation between the two acquisitions, improving the accuracy of any derived quantities (27, 28).

To our knowledge, only limited information is available in the literature regarding comparison of contrast and noise between DS-DECT MEIs and conventional 120-kVp images for pancreatic cancer patients. Hardie et al. (18) compared DS-DECT 55 keV MEIs to simulated linearly blended 120-kVp images but did not compare them to conventional 120-kVp images. The study also did not examine other levels of MEIs. Noid et al. (19) compared SE-DECT MEIs to conventional 120-kVp CT images, but did not analyze DS-DECT MEIs.

Similarly, there is limited information regarding metal artifact reduction through DS-DECT and metal artifact-reducing algorithms. The aforementioned study by Zhou et al. (9) compared a range of DECT MEIs to simulated weighted 120-kVp images but did not compare them to conventional 120-kVp images, nor did the study investigate metal artifact-reducing algorithms in conjunction with MEIs. Yue et al. (20) compared a range of DECT MEIs enhanced by a metal artifact-reducing algorithm but did not compare the MEIs to conventional 120-kVp images. The study also used a limited range of MEI energy levels (between 80 and 140 keV) (20).

Similarly, there is limited information regarding motion artifact reduction through DECT. A recent study examined the motion artifact reduction from SE-DECT to DS-DECT for kidney stone patients and analyzed it by measuring the dimensions of kidney stones (29). However, to our knowledge, motion artifact reduction has not been examined in the context of radiation oncology.

The purpose of this work is to investigate the advantages of DECT for tumor and OAR delineation during external beam RT planning. We will demonstrate a variety of cases in which DECT protocols can offer additional value to the planning process by mitigating image artifacts, reducing registration uncertainty, and enhancing image features for more accurate segmentation. We will offer novel contributions to the literature by comparing a wide range of DS-DECT MEIs to conventional 120-kVp CT images for contrast and noise, using pancreatic cancer cases. We will compare a range of DS-DECT MEIs to conventional 120-kVp CT images for metal artifact reduction, incorporating metal artifact-reducing algorithms.

## Materials and Methods

This retrospective, single-center, data analysis was approved by the MCW Institutional Review Board. The need for written informed consent was waived due to the retrospective nature of the study.

CT datasets were acquired for 20 representative patients of different tumor sites and/or clinical situations in the routine RT simulation using both SE-DECT enabled scanner (Definition AS Open, Siemens) and DS-DECT scanner (Drive, Siemens). The dataset includes 11 pancreas cases, two liver cases, a palliative T spine case, a prostate case, a large mantle case, a breast case, a thymus case, a tonsil case, and a meningioma case. The datasets were then analyzed to demonstrate various advantages of DECT for RT planning.

The SE-DECT data were acquired using a protocol known as Dual Spiral Dual Energy (30). The protocol generated two IV contrast-free CT datasets through successive helical scans at different kV and mA levels but with the same tube rotation time: 0.5 s. The first and second scans were set at 80 and 140-kVp (no Sn filter was available) with a pitch of 0.6 and 1.2, respectively. The field of view (FOV) was 500 mm by 500 mm. Imaging dose was controlled by an automatic exposure controlling (AEC) algorithm (CareDose4D). The purpose of an AEC is to match the image quality of reference scans based on the diameter of a patient as measured in the scout image (31, 32). Thus, the dose was varied from patient to patient for image quality preservation purposes, but the combined reference dose level was set equal to the dose for a standard abdominal CT [volume computed CT dose index (CTDIvol = 16.99 mGy)]. SE-DECT was then reconstructed with a medium sharp convolution kernel (I30f) through an iterative reconstruction (SAFIRE strength 5; Siemens Healthcare) to reduce noise without diminishing spatial resolution (19, 33).

The DS-DECT data were acquired using a DS scanner operating in DECT mode. In this method, two sinograms were simultaneously acquired using two X-ray tubes and two detectors separated by 95° in the same gantry. The “A” tube was set to scan at 80-kVp, while the “B” tube was set at 140-kVp with a tin filter added. Total reference dose level was 20 mGy and pitch was 0.6; patients received IV bolus when indicated. Unless stated otherwise, voxel size was 0.98 mm × 0.98 mm × 2 mm (1 mm in the scanning plane with a 2-mm slice interval). The selected iterative reconstruction was Admire strength 3 and reconstruction kernel was I30f. Standard FOV of the “A” tube was 500 mm, while the “B” tube FOV was 330 cm. Thus, all derived study sets had a FOV of 330 mm.

Conventional CTs were acquired for comparison using the DS-DECT scanner operating with the “A” tube alone. Conventional scans were performed at a tube potential of 120-kVp, pitch of 0.6, tube rotation time of 0.5 s, CTDIvol of 20 mGy, and a scan resolution of 0.98 mm × 0.98 mm × 2 mm. The raw data were reconstructed using a medium sharp convolution kernel (I30f).

Dual-energy CT data were used to derive a variety of images. Virtual MEIs were generated from 40 to 190 keV in 1 keV increments. For CT sets acquired with iodine contrast, I map (iodine quantified in each voxel) images were generated. Fat maps were also generated where indicated. The data for each of the study sections were collected and analyzed as follows:

### Reduction in Metal Artifacts

Conventional CT (120-kVp) and DS-DECT scans were acquired for a spine case involving spinal implants and a prostate case involving bilateral hip replacements. DS-DECT scans were used to generate MEI study sets. All study sets were subsequently processed with a metal artifact-reducing algorithm (iMAR, Siemens Healthineers) (34). The MEIs with and without iMAR were then compared to the conventional CTs with and without iMAR to evaluate the effect of metal artifact reduction.

### Reduction in Photon Starvation

Conventional CT and DS-DECT scans were acquired for a representative large patient who was unable to lift his arms above his head during simulation. DS-DECT scans were used to generate MEI study sets. The MEIs and conventional CTs were then compared for CT intensity and, thus, the photon starvation effect.

### Contrast Enhancement

Conventional CT data and DS-DECT were acquired for 10 patients with pancreatic cancer. All cases had undergone RT stimulation with gastric and IV bolus and had been scanned using 120-kVp CT during the late arterial phase followed by a DS-DECT scan. DS-DECT scans were used to generate MEI and I map study sets. The pancreatic head and duodenum were delineated in the conventional CT and DS-DECT scans, and image contrast between the two structures was calculated and compared. Image contrast was defined as the difference in mean CTN between the pancreatic head and duodenum. The contrast-to-noise ratio was similarly calculated as image contrast divided by the average of the standard deviation of mean CTN in the two structures. To further demonstrate image contrast enhancement across a range of treatment sites, data for three representative cases with breast (invasive ductal carcinoma), liver (common bile duct cholangiocarcinoma), and thymus (thymic carcinoma) cancers were analyzed. The breast case was IV-bolus free, while the liver and thymus cases were scanned with iodine contrast using the hepatic phase and a set 25-s delay, respectively.

### Mapping Vasculature

Dual-source DECT, conventional 120-kVp CT, and PET scans were acquired for a representative patient with squamous cell carcinoma in the left tonsil; the patient received IV bolus with a set 40-s delay. The DECT scans were acquired immediately after the conventional CT scans. PET and conventional CT scans were fused by a rigid-body registration, and an I map was generated from the DS-DECT scan. Tumor volume was reconstructed based on the I map and then compared to the volume delineated from the PET/CT fusion.

### Reduction in Motion Artifacts

Simultaneous DECT (e.g., DS-DECT) and SE-DECT free breathing scans were acquired for a pancreatic cancer patient with involvement of the celiac artery. MEIs were derived from the simultaneous and sequential scans after which the celiac artery was delineated. Dimensions of the two celiac artery contours were then compared to quantify the blurring of spatial resolution in the SE-DECT.

### Fat Maps

Dual-source DECT data were acquired from a representative meningioma patient and used to generate MEIs and fat maps for comparison to MRI (T1 plus contrast). Image contrast between the tumor and surrounding cerebral tissue was then compared for each of the study sets. Fat maps are currently only FDA-approved for liver scans.

## Results

The results of our investigations are broken down into the different categories as described in section “Materials and Methods.” Since a purpose of the study was to compare DECT to the conventional CT for RT planning, data were obtained to compare DECT MEI and CT for metal artifact reduction, photon starvation effect, image contrast, and vasculature mapping. These results are presented here. In addition, results are presented for the evaluation of simultaneous DECT vs. SE-DECT, as well as for the study on DECT-derived fat maps in the context of DECT-derived MEIs and T1 + contrast images.

### Reduction in Metal Artifacts

**FIGURE 1:**
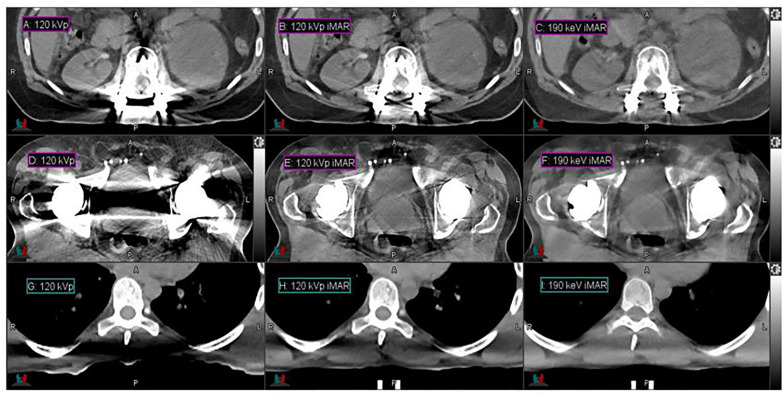
Details from the axial slices of a palliative spine cancer patient with metal implants imaged with 120-kVp **(A)**, 120-kVp iMAR **(B)**, 190 keV iMAR **(C)**; a prostate cancer patient with dual hip replacements imaged with 120-kVp **(D)**, 120-kVp iMAR **(E)**, and 190 keV iMAR **(F)**; a large mantle field patient imaged with 120-kVp **(G)**, 120-kVp with eFOV **(H)**, and 190 keV MEI **(I)**. For all images, the window width is 400 HU and level is 40 HU.

High-energy MEIs derived from DECT offer substantial reductions in metal artifacts compared to conventional CT images, as demonstrated in Figure 1. Low- and high-intensity streaking artifacts are most evident in the conventional 120-kVp scans without iMAR (Figures 1A,D). Applying iMAR to the conventional CT images reduces the severity of the artifacts but does not eliminate them (Figures 1B,E). The greatest metal artifact reduction was obtained by using MEIs at the highest energy (190 keV) combined with iMAR (Figures 1C,F). These 190 keV images with iMAR (Figures 1C,F) demonstrated a metal artifact reduction of over 95% compared to conventional CTs (Figures 1A,D), as calculated from the volume of low CTN voxels restored. It should be noted that the metal artifact reduction observed presently from DS-DECT can be achieved via any DECT acquisition protocol.

### Reduction in Photon Starvation

High-energy MEIs derived from DECT offer substantial reductions in photon starvation compared to conventional CT images, as demonstrated in Figure 1. Figures 1G–I present the CTs acquired for the representative large patient. In the conventional 120-kVp scans (Figures 1G,H), a photon starvation region of 367 cm^3^ with a degraded CT intensity is seen near the spine. However, high-energy MEIs eliminate the degradation and restore the image quality, as calculated from the volume of low CTN voxels restored. The 190 keV MEI (Figure 1I) demonstrates the greatest reduction in photon starvation among all MEIs.

### Image Contrast Enhancement

**FIGURE 2:**
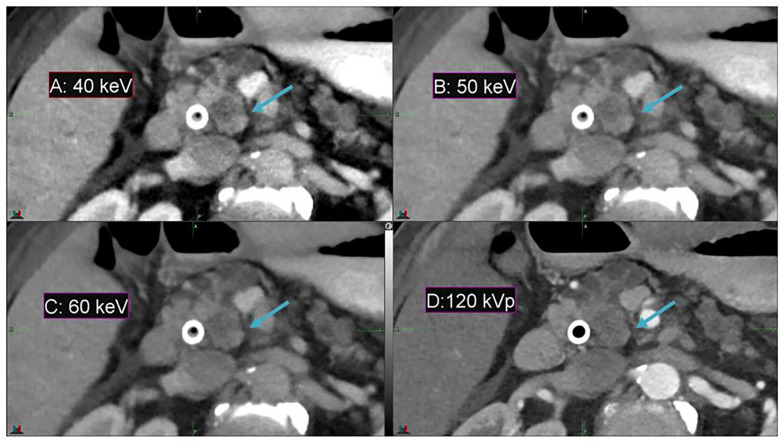
Details from an axial slice of a pancreatic cancer patient imaged with 40 keV MEI **(A)**, 50 keV MEI **(B)**, 60 keV MEI **(C)**, and 120-kVp **(D)**. The window widths and levels are 550 HU, 120 HU **(A)**; 430 HU, 100 HU **(B)**; 405 HU, 80 HU **(C)**; and 400 HU, 50 HU **(D)**. The pancreatic tumor is indicated by the arrow.

In comparison to conventional CT, DECT offers a substantial enhancement in image contrast between the duodenum and pancreatic head in the pancreas patient study sets, as demonstrated by Figure 2. Contrast enhancement is maximized at the lowest-energy MEI (40 keV, Figure 2A).

**FIGURE 3:**
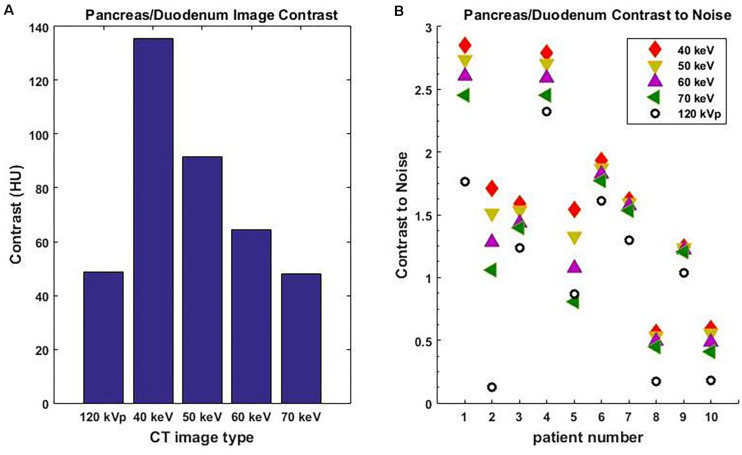
Average image contrast **(A)** for 10 pancreatic cancer patients at 120-kVp, 40, 50, 60, and 70 keV. Contrast-to-noise ratio **(B)** for those patients at 120-kVp, 40, 50, 60, and 70 keV (*n* = 10, *p* = 0.002, Student’s *t*-test).

Among all 10 pancreatic cancer patients, the average image contrast at the conventional 120-kVp CT is 48.9 ± 38.0 HU, while the average image contrast at 40 keV is 135.5 ± 125.1 HU, an increase by a factor of 2.8 (Figure 3A). For every case analyzed, the image contrast is maximized at 40 keV. Please note that the variance in these calculations is largely due to inter-patient variation in IV and gastric bolus intake.

It is well known that noise increases rapidly as the MEI energy decreases. However, the contrast also increases as MEI energy decreases. As a result, the contrast-to-noise ratio is a useful measure of the relative quality of images. For all 10 pancreatic cancer patients analyzed, the contrast-to-noise ratio was maximized at 40 keV due to the dramatic enhancement in image contrast (Figure 3B). The difference in contrast-to-noise ratio between 120 and 40 keV for the 10 patients was statistically significant as calculated using the paired Student’s *t*-test (*p* = 0.002). Note that a 70 keV MEI has similar image contrast to a 120-kVp CT because the mean X-ray energy from a 120-kVp CT is around 70 keV.

**FIGURE 4:**
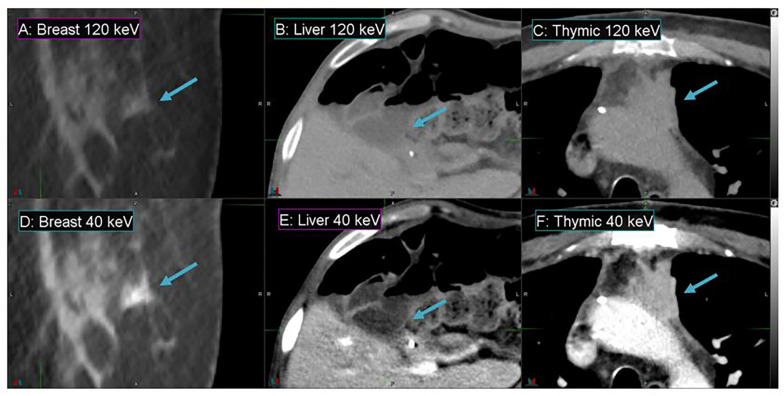
Details from axial slices of breast **(A,D)**, liver **(B,E)**, and thymic **(C,F)** cancer patients imaged with 120 keV MEI **(A–C)** and 40 keV-MEI **(D–F)**. The window width is 400 HU for all images. The window levels of **(A–C)** are 40 HU, and those of **(D–F)** are 100 HU, 140 HU, and 90 HU, respectively. The tumors are indicated by the arrows.

**FIGURE 5:**
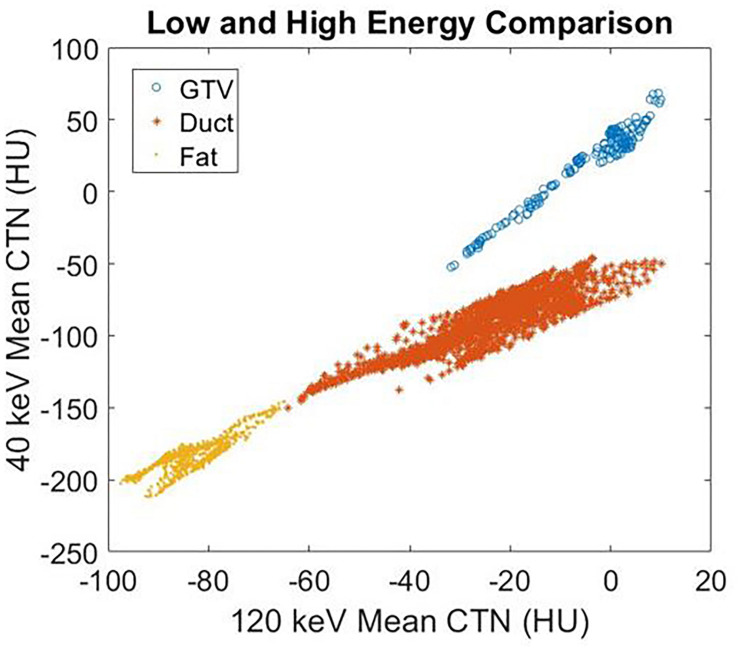
The mean CTN at 40 and 120 keV for the GTV, ductal tissue, and fat regions of the breast cancer patient in Figure 4.

Figure 4 demonstrates the contrast enhancement for three other representative patients on DS-DECT. The first case (Figures 4A,D) had an invasive ductal carcinoma in the left breast treated with pre-operative RT in the prone position. The second case (Figures 4B,E) had an intrahepatic bile duct carcinoma, and the third case (Figures 4C,F) had a thymic carcinoma with aortic invasion. Clearly, the tumors (indicated by arrows) are more visible at 40 keV than at 120 keV. To illustrate the enhancement of tumor visibility for the breast cancer patient, the mean CTN was calculated for the regions corresponding to the tumor, ductal tissue, and fat at both 40 and 120 keV (Figure 5). The tumor regions were clearly defined from the other regions by mean CTN.

### Mapping Vasculature

**FIGURE 6:**
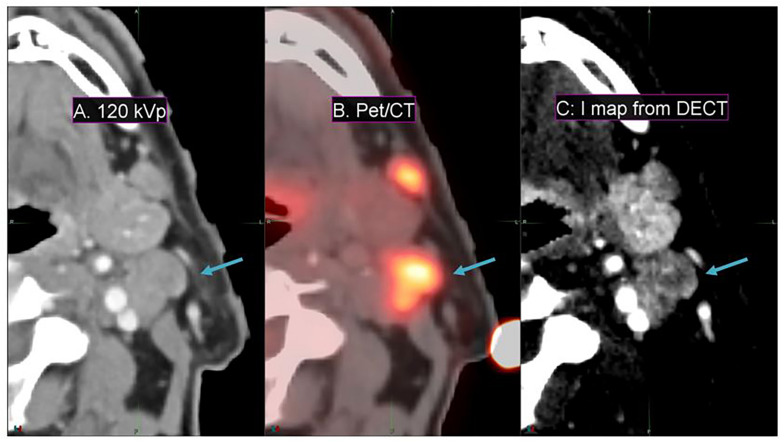
Details from an axial slice of a tonsil squamous cell carcinoma patient imaged with 120-kVp **(A)**, PET/CT **(B)**, and I map derived from a DECT **(C)**. The window width is 400 HU and level is 40 HU. The tonsil tumor is indicated by the arrow.

Conventional 120-kVp CT (Figure 6A) of a head and neck squamous cell carcinoma patient shows little enhancement in the PET active region, compared to the spatial correlation exhibited between the PET active and I map regions (Figures 6B,C) of the same patient. This implies that DECT-derived I maps can emphasize vasculature that correlates with pathological information typically extracted from functional or biological imaging (for instance, PET/CT) that cannot be found in conventional CT.

### Reduction in Motion Artifacts

**FIGURE 7:**
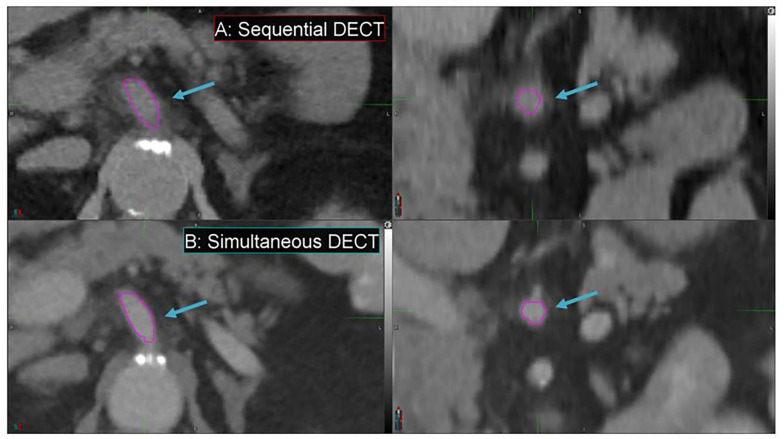
Details from the axial and coronal slices of a pancreatic cancer patient imaged with 70 keV MEI, derived from a sequential DECT **(A)** and a simultaneous DECT **(B)**. A magenta region of interest was drawn to highlight the celiac artery. The window width is 400 HU and level is 40 HU.

Simultaneous DECT offers substantial improvements in enhancing spatial resolution and reducing motion artifacts compared to SE-DECT, thus improving image quality, as evident in a pancreatic cancer patient study set (Figure 7). Due to breathing and/or peristalsis motion between the two acquisitions in the sequential protocol, the celiac artery appears 3 mm larger in diameter in the SE-DECT image than in the simultaneous DECT image (Figures 7A vs. B). Furthermore, this motion-related blurring is evident throughout the SE-DECT image, in contrast to the clearer simultaneous DECT image.

### Fat Map

**FIGURE 8:**
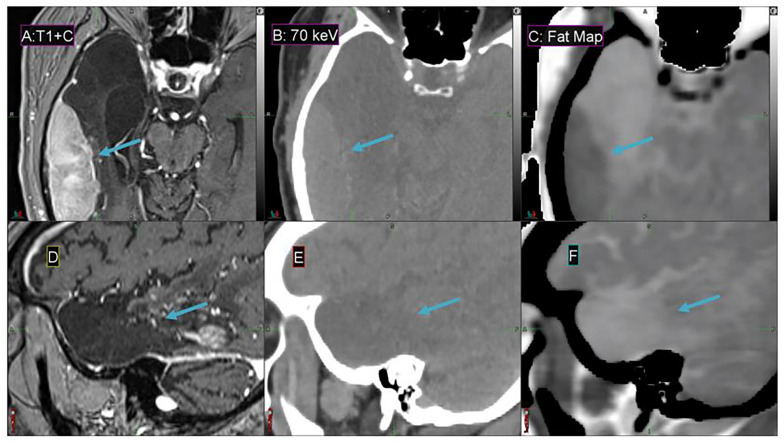
Details from the axial **(A–C)** and sagittal **(D–F)** slices of a meningioma patient imaged with T1 + contrast MR **(A,D)**, 70 keV MEI **(B,E)**, and a fat map **(C,F)** derived from a simultaneous DECT. The window width is 400 HU and level is 40 HU for **(B,E)**. The meningioma tumor is indicated by the arrow.

Fat maps derived from DS-DECT (while not clinically approved for this application) demonstrate a substantial contrast enhancement between the tumor region and adjacent cerebral tissue for the representative meningioma patient, in comparison to associated MEIs and T1 + contrast images (Figure 8). The 70 keV MEI slices (Figures 8B,E) show relatively poor contrast compared to the corresponding fat map slices (Figures 8C,F).

## Discussion

Dual-energy CT enables the derivation of study sets that provide clinical information, which is either obscured by image artifacts or inherently missing in conventional CT. Our study demonstrated these advantages, which include greatly enhanced soft tissue contrast from low-energy MEIs, improved visualization of vasculature with I maps, restored image quality in cases of photon starvation, metal artifact reduction from high-energy MEIs, and motion artifact reduction with DS-DECT compared to SE-DECT.

Furthermore, the derived study sets can be obtained without sacrificing conventional CT study sets or exposing the patient to additional dose. Simultaneous DECT can be used to derive these study sets, offering substantial improvements in spatial resolution and motion artifact reduction compared to SE-DECT. For instance, in our study, simultaneous DECT study sets showed a 3-mm reduction in celiac artery diameter in our pancreatic cancer case compared to SE-DECT study sets. The smaller diameter in the simultaneous DECT image signifies a more accurate measurement of the artery’s size. The larger diameter in the SE-DECT image is due to motion blurring.

Based on this study, patients that receive bolus during simulation (IV or gastric) may benefit from the generation of I maps. Typically, this includes pancreas, liver, and head and neck cancers. Furthermore, low-energy MEIs derived from DECT will enhance soft tissue contrast at these sites even in the absence of bolus. A previous study by Hardie et al. (18) examined pancreatic lesion contrast-to-lesion ratio for pancreas patients with bolus and observed that 55 keV MEIs had a significantly higher contrast-to-noise ratio (6.8 ± 4.1) compared to simulated linearly blended 120-kVp images (5.8 ± 3.8; *p* = 0.0002). Our study expands on Hardie et al.’s (18) work by comparing DS-DECT-derived MEIs ranging from 40 to 190 keV to conventional 120-kVp CT. We found that 40 keV MEIs display a higher contrast-to-noise ratio than not only the conventional 120-kVp CT (greater by a factor of 2.8) but also the 55 keV MEI. Another previous study of pancreas patients without bolus showed that the SE-DECT protocol offers a contrast enhancement of more than a factor of two (19), offering similar results to our study for the SE-DECT option.

In the current study, high-energy MEIs have been demonstrated to restore image quality to study sets degraded by photon starvation due to patient size. In addition, 190 keV can be used to generate whole-body patient contours using a standard thresholding method. If a conventional CT is being used for planning, the whole-body contour from the 190 keV MEI can be transferred after a rigid registration is performed.

High-energy MEIs can also work in concert with metal artifact-reducing reconstructions to reduce image streaking associated with high Z materials in the FOV. In a previous study, Yue et al. (20) compared MEIs at 80, 100, 120, and 140 keV with and without metal artifact reduction software. The study found that 120 and 140 keV MEIs with metal artifact reduction software offer the best image quality (20). Our study expands on these results, as our highest-energy MEIs (190 keV) with iMAR offered the best image quality (as calculated from the volume of low CTN voxels restored) compared to lower-energy MEIs and conventional 120-kVp CT.

Improving image quality via DECT will positively impact RT in the clinic. For example, enhanced CT image quality has been shown to improve image registrations (35), particularly deformable registrations used in RT (36). In the purview of CT-based RT simulation and planning, multiple images are often acquired, and image registrations are therefore necessary. Using DECT will reduce artifacts and improve contrast in these images, which will then improve the quality of RT plans by reducing uncertainty in image registration as well as inter- and intra-observer variations during structure delineation. Because enhanced image quality reduces the uncertainty inherent in soft tissue registrations (35), daily patient setup precision for fractionated RT will also be improved. Furthermore, reducing CT artifacts is anticipated to increase dose calculation accuracy. This is a topic of our ongoing research.

In addition to enhanced image quality and improved image registration, DECT holds more clinical advantages for IGRT. In IGRT, the daily image is registered with the planning image, even if the daily image (e.g., cone-beam CT) has a different image quality from the planning image. The registration will be optimized if the daily image has the same quality as the planning image, further supporting the use of DECT for IGRT if DECT is used for planning.

Higher image quality can also positively impact treatment planning by enabling more accurate delineation of volumes. With reduced delineation uncertainty, the margin introduced to account for the uncertainty will be reduced, resulting in smaller planning target and/or organ-at-risk volumes. This, in turn, facilitates the achievement of desired dosimetric constraints, allowing for improved treatment plans that maximize coverage of the target regions while minimizing exposure to OAR.

Another clinical advantage is that the use of DECT does not require changes to the workflow used for conventional CT for simulation and planning. Outside of the specific CT acquisition protocol, DECT-based simulation is identical to the conventional simulation process. Because the DECT data (e.g., MEI) required for treatment planning is generated automatically, there is also no change to the treatment planning workflow for the planners.

While DECT does not require a change in RT workflow, we would like to point out that other imaging modalities (e.g., MRI) may be used to address some of the issues we discussed (e.g., soft tissue contrast). However, such additional modalities may not always be available; they may not be acquired with the patient in the same treatment position, which inevitably requires multi-modality image registration. This potentially introduces additional uncertainty into the planning process. On the other hand, derived images from DECT face none of these limitations.

Admittedly, there are limitations to DECT-based simulation. Derived images are only available in the region where the fields of view of the two acquisitions overlap. For the DS-DECT scanner used in this study, the overlapping area was limited to a cylindrical region measuring 330 mm in diameter. In addition, DECT acquisitions were not available in cases that required respiratory gating or where a 4D study set was necessary. The only motion management that could be applied was acquiring the scans during breath-hold, which may not be possible for all patients. It is also necessary to be aware that CTN in derived images may differ from those in a conventional 120-kVp image. Hence, when calculating the dose to the patient, dosimetrists must employ the appropriate CT-to-electron density curve to avoid inaccurate dosimetry. However, recent literature demonstrates that DECT can be used to derive relative electron density images, eliminating the need for applying a CT-to-electron density curve (14). Finally, there may be concerns over additional patient dose since DECT is inherently two CT acquisitions. However, the imaging dose from the two individual scans that make up the DECT is designed to add up to a comparable imaging dose of a conventional CT protocol.

## Conclusion

Dual-energy CT offers considerable advantages over conventional CT for RT planning. DECT provides unique powerful post-processing capabilities that can substantially improve image contrast, reduce metal artifacts, and reduce photon starvation. In addition, DECT-derived image sets, such as iodine maps and fat maps, can highlight certain normal structure and/or tumor features. Simultaneous DS-DECT can minimize the motion artifact compared to other DECT methods. All these advantages will greatly improve tumor and normal structure delineation for RT planning, suggesting that DECT may serve as the future standard of RT simulation.

## Data Availability Statement

The raw data supporting the conclusions of this article will be made available by the authors, without undue reservation.

## Ethics Statement

This retrospective, single-center, data analysis was approved by the MCW Institutional Review Board. The need for written informed consent was waived due to the retrospective nature of the study.

## Author Contributions

GN: study design, data collection, data analysis, and manuscript drafting. JZ: data collection, literature search, formatting, and manuscript editing. AT: scan protocols design and manuscript editing. NM: technical support of scan protocols and post processing. DS: data analysis and editing. DP and EP: design of scan protocols and editing. CS: inputs on clinical issues and editing. XL: study design, project management, and manuscript editing. All authors contributed to the article and approved the submitted version.

## Conflict of Interest

NM was employed by the company Siemens Healthineers. The remaining authors declare that the research was conducted in the absence of any commercial or financial relationships that could be construed as a potential conflict of interest.
